# Miscellany

**Published:** 1893-08

**Authors:** 


### Miscellany.

---

THE PAN-AMERICAN MEDICAL CONGRESS EXCURSION TO ROME.—It has been definitely determined that the Pan-American Medical Congress Excursion to the Eleventh International Medical Congress will sail on the S. S. "Werra," from New York, September 9th, the day following the adjournment of the Congress at Washington, and will arrive at Genoa, September 20th, four days before the opening of the Rome meeting.

Round-trip steamer tickets may be procured for $142.50 for inside rooms, and $150.00 and upwards for outside rooms. Tickets are good for members of the Congress and their families, and may be used at option of holder, to return on any steamer of the line from Geno, or on Saturday steamers from Bremen, or Sunday steamers from Southampton, during the months of October, November, and December. Physicians desiring to avail themselves of this exceptionally low rate should at once become members of the Pan-American Medical Congress by sending the registration fee ($10.00) to the Treasurer, Dr. A. M. Owen, Evansville, Ind., and informing the Secretary-General, Dr. Chas. A. L. Reed, Cincinnati, of their intention to join the excursion. Passage should be secured without delay, as the trip, involving, as it will, a stop at the Azores and Gibraltar and a sixty hours' sail along the picturesque coasts of Spain, France, and Italy, promises to be very popular. Many prominent European guests of the Pan-American Congress will return on this occasion. The time allowed will afford American physicians an opportunity to not only attend the International Congress and visit Rome, but to extend their journey to the famous sanatoria of South France and the Riviera.

---

Official Delegates to the Pan-American Medical Congress. —Practically all of the governments have appointed official delegates to the Congress in response to the invitation by the President of the United States. The United States Government will be represented by six delegates. The larger cities of all the Latin-American countries have appointed delegates to participate in the proceedings of the Sections on Hygiene, Climatology, and Demography, and on Marine, Hygiene, and Quarantine, and similar appointments will be made by the cities of the United States. Seventy-six similar delegates have, so far, been appointed by the governors of States in the United States. A large number of delegates have been chosen by the medical colleges of the United States and other American countries to attend the Section on Medical Pedagogics, under the presidency of Professor J. Collins Warren, of Boston.

---

Section on Materia Medica and Pharmacology, Pan-American Medical Congress.—A Section on Materia Medica and

Pharmacology has been organized under the executive presidency of Prof. Joseph P. Remington, of Long Port, N. J., with Prof. F. G. Ryan, 3739 Brown street, Philadelphia, as English-speaking secretary. This Section promises to be one of the most important of the entire Congress. Delegates have been invited from all the pharmaceutical societies and colleges in all the Americas. Those contemplating attendance are invited to prepare papers on pharmaceutical topics. Titles should be sent at once to Professor Ryan, Secretary.

---

Dr. Ernst Hart, editor of the *British Medical Journal*, and Prof. Dr. Czerny, of Heidelberg, will be among the distinguished guests of the Pan-American Medical Congress. The latter is booked for the Pan-American excursion to Rome by the "Werra."

---

The Sterilization of Ophthalmic Instruments.—According to the *Medical Press and Circular*, M. Nuel, of Liège, France, has reported on the above subject, giving the results of his personal researches. He has found that the use of boiling water has served him as the surest and readiest method in regard to the major part of his instruments. The antiseptic efficacy of thus plunging the articles into boiling water is enhanced if to the water is added some potash or soda, or even common salt. The recommendation of Schimmelbusch is referred to, namely, to use a solution of carbonate of soda in a strength of one and one-half to two per cent., and this solution is suitable to be used in the cleansing of instruments. The period of immersion may be three or four seconds in the case of cutting instruments, while it should be prolonged to thirty seconds for needles, forceps, and the like. The reporter has used a variety of other recommended measures of sterilization, but has given the preference to the boiling water method.—*Journal American Medical Association.*

